# The effect of oxytocin nasal spray on social interaction in young children with autism: a randomized clinical trial

**DOI:** 10.1038/s41380-022-01845-8

**Published:** 2022-10-27

**Authors:** Adam J. Guastella, Kelsie A. Boulton, Andrew J. O. Whitehouse, Yun Ju Song, Rinku Thapa, Simon G. Gregory, Izabella Pokorski, Joanna Granich, Marilena M. DeMayo, Zahava Ambarchi, John Wray, Emma E. Thomas, Ian B. Hickie

**Affiliations:** 1 Clinic for Autism and Neurodevelopment (CAN) Research, Brain and Mind Centre, Children’s Hospital Westmead Clinical School, Faculty of Medicine and Health, University of Sydney, Camperdown, NSW Australia; 2 Telethon Kids Institute, University of Western Australia, Perth, WA Australia; 3 Department of Neurology, Duke University School of Medicine, Durham, NC USA; 4 Child and Adolescent Health Service, Child Development Service, West Perth, WA Australia

**Keywords:** Autism spectrum disorders, Neuroscience

## Abstract

Early supports to enhance social development in children with autism are widely promoted. While oxytocin has a crucial role in mammalian social development, its potential role as a medication to enhance social development in humans remains unclear. We investigated the efficacy, tolerability, and safety of intranasal oxytocin in young children with autism using a double-blind, randomized, placebo-controlled, clinical trial, following a placebo lead-in phase. A total of 87 children (aged between 3 and 12 years) with autism received 16 International Units (IU) of oxytocin (*n* = 45) or placebo (*n* = 42) nasal spray, morning and night (32 IU per day) for twelve weeks, following a 3-week placebo lead-in phase. Overall, there was no effect of oxytocin treatment over time on the caregiver-rated Social Responsiveness Scale (SRS-2) (*p* = 0.686). However, a significant interaction with age (*p* = 0.028) showed that for younger children, aged 3–5 years, there was some indication of a treatment effect. Younger children who received oxytocin showed improvement on caregiver-rated social responsiveness ( SRS-2). There was no other evidence of benefit in the sample as a whole, or in the younger age group, on the clinician-rated Clinical Global Improvement Scale (CGI-S), or any secondary measure. Importantly, placebo effects in the lead-in phase were evident and there was support for washout of the placebo response in the randomised phase. Oxytocin was well tolerated, with more adverse side effects reported in the placebo group. This study suggests the need for further clinical trials to test the benefits of oxytocin treatment in younger populations with autism.

Trial registration www.anzctr.org.au (ACTRN12617000441314).

## Introduction

Autism spectrum disorder (autism) is a neurodevelopmental condition characterized in the DSM-V by impairments in social communication and interaction, and the presence of stereotypical and repetitive behaviors [1]. The current incidence of autism within the general population is estimated at one in 44 children [2]. While psychotropic drugs, such as risperidone, are often used to manage irritability and behavioral problems, there is no evidence for efficacy of these medications for social communication, learning, and responsiveness [3]. While behavioral interventions have been found to improve social interaction and responsiveness [4], they are often time-consuming and costly to implement [5, 6]. There are, therefore, limited supports for autistic children targeting social learning and development.

Oxytocin is a crucial regulatory hormone to both early life social learning and lifelong social behavior [7, 8]. In humans, administration of oxytocin has been found to improve a range of outcomes associated with social responsiveness, including eye gaze [9], emotion recognition [10, 11], social cognition and neural circuitry associated with social cognition [8]. Several studies have investigated the potential benefits of oxytocin to children with autism. In young children, there have been mixed results regarding efficacy of oxytocin [12]. Two studies have suggested that twice-daily administration improves social responsiveness in autistic children [13, 14]. These trials administered oxytocin or placebo morning and night for a period of 4 to 5 weeks. In contrast, a third trial administering oxytocin or placebo four times, in total, showed no benefit [15]. Most recently, a large study across 277 participants aged 3–16 showed no benefit of oxytocin relative to placebo [16]. Across these studies, intranasal administration has been the preferred route of administration due to it being well-tolerated and easy to use. Oxytocin is also often administered intranasally as this route is thought to penetrate the brain and produce behavioral effects [17]. These studies have, however, not been without controversy. Reviews of this evidence have highlighted the potential for placebo and expectancy effects to impact outcomes [14, 18, 19], a need for biological markers [13], a better understanding of drug delivery factors to inform use [20], and the role of early life development and social learning contexts [8], all of which might potentially moderate response [12]. To illustrate, the age of the child may play a part, with some evidence suggesting that younger children may show greater response to social interventions [21]. Other research has highlighted that oxytocin responses may be partly moderated by placebo effects [14, 18, 19]. There is also a need to further establish evidence around oxytocin use in social development to inform ethical and scientific arguments about whether it remains a sound target for intervention to support outcomes for autistic people.

This present study investigated the efficacy, tolerability, and safety of intranasally-administered oxytocin to improve social interaction difficulties in young autistic children. This study included a placebo lead-in phase to evaluate response to drug to characterize potential responders and focused recruitment at a younger age than most clinical trials. We predicted that oxytocin nasal spray would be both safe and tolerable for this cohort of young autistic children. We further predicted that oxytocin would improve caregiver-reported social responsiveness, and a clinician rating of improvement in social behavior in young children with autism, and that this effect would be largest in younger children.

## Materials and methods

### Study design

Patients were enrolled in a double-blind, randomized controlled, placebo lead-in, multi-site trial of oxytocin nasal spray and an identical placebo across four assessment time points (wk 0 baseline, wk 3 post-placebo lead in, wk 15 post randomized treatment, wk 27 follow-up assessment). The study was conducted at the Brain & Mind Center (BMC), University of Sydney, and the Telethon Kids Institute (TKI), Western Australia. Ethical approval was provided by the University of Sydney Human Research Ethics Committee (2013/502). The trial was pre-registered prior to recruitment with the Australian Clinical Trials Registry (ACTRN12617000441314). Informed consent from caregivers was obtained for each participant.

### Participants

Children aged between 3 and 12 years of age who met DSM-5 criteria for Autism Spectrum Disorder were recruited. Children aged between 3–12 were recruited to the BMC, while children aged 3–6 were recruited to TKI. This decision was based on available funding provided to each site. Participants were recruited through advertisements and specialist networks. To confirm eligibility, caregivers of participants initially completed a telephone screening assessment to determine whether their child had received a previous diagnosis associated with autism and were not likely to meet noted exclusion criteria.

Participants then completed screening assessments, the Autism Diagnostic Observation Schedule, Second Edition (ADOS-2) [22], an intelligence assessment if one had not been conducted in the last two years (The Leiter International Performance Scale-Revised [23]) and a medical interview. Exclusion criteria included known sensitivity to preservatives in the nasal spray (in particular, Benzyl Alcohol). All participants were stabilized on psychotropic medication for 8 weeks before commencement of the trial, and no changes to dose were made for the duration of the trial. Participants were asked not to change current treatment regimes, such as behavioral therapies, for the duration of the trial.

### Medication

Oxytocin nasal spray consisted of 8IU each spray per nostril morning and night; 32IU per day). The placebo spray included all of the same ingredients except oxytocin. All sprays contained sorbitol (2%), glycerol (2%), benzyl alcohol as preservative, and distilled water, within an amber 8 ml glass nasal spray with metered Pfeiffer pump spray bottle. Drug kits were manufactured by PCI Pharma Services. We followed guidelines outlined by both Guastella et al., 2013 [17], and GMP manufacturing guidelines in relation to both manufacture and stability testing. Nasal sprays for Visit 2 were labeled with sequential numbers corresponding to order of entry into the trial and stratified by gender by the trial pharmacist. At Visit 1, participants were allocated a placebo spray for 3 weeks using a single-blinded schedule. At Visit 2, participants were randomly assigned drug kits containing either oxytocin or placebo (which included identical ingredients except oxytocin) in a double-blinded schedule. Blocking was in sets of six (three active and three placebo) in a randomly generated order. All research staff conducting assessments, as well as caregivers and participants, were blinded to condition allocation and unaware of randomization at Visit 2. The drug kits for each treatment (oxytocin and placebo) provided at Visit 2 contained two nasal spray bottles to be administered over the course of 12 weeks.

### Assessment schedule

A schedule of assessments conducted at each visit is provided in Supplementary Table 1. Following informed consent by the caregiver, eligible children visited in wk 0 to complete assessments and receive placebo nasal spray (study baseline, Visit 1). All participants were informed that they were being randomly allocated to either oxytocin or placebo after Visit 1. All participants were, however, assigned to receive a placebo spray for three weeks in a placebo lead in phase. Participants returned to complete Visit 2 assessments and drug randomization (wk 3). The placebo lead-in phase used a single-blind approach, with assessors aware of the treatment condition (placebo). The 12-week intervention phase used double-blinding, with both assessors and participants blind to treatment condition (oxytocin or placebo). Following the 12-week intervention, participants returned at wk 15 to complete assessments (Visit 3). Three months after the intervention, participants returned at wk 27 to complete follow-up assessment (Visit 4). Site visits, training, and regular online meetings were conducted to establish reliability across assessments and sites, as well as to review cases. Assessors across sites also met via videoconference regularly to ensure uniformity in delivery of the trial.

### Medication schedule and adverse event reporting

Instructions were provided to caregivers on site during drug allocation visits (Wk 0 and 3) consistent with our previous published guidelines [17]. Caregivers were instructed to administer the nasal spray in the morning and evening. It was recommended that the nasal spray be administered before breakfast and before dinner, as children were asked to abstain from food and drink other than water for two hours before receiving the nasal spray. In addition, caregivers were given a drug diary and an information pack on nasal spray delivery and symptom monitoring.

Monitoring of potential adverse events was by telephone during mid-intervention for the treatment phase (i.e., wk 9) and at treatment completion (wk 15). Caregivers (and the child when appropriate) were asked open-ended questions about adverse events or side effects that had occurred during the treatment period. Caregivers were also asked to complete a daily diary reporting any side effects of nasal spray administration. This diary was also used to evaluate treatment compliance and was reviewed during Visit 3. Serious adverse events were reported to the local institutional review board.

### Primary and secondary outcome measures

#### Primary outcomes

The first primary outcome measure was the caregiver-completed Social Responsiveness Scale, Second Edition (SRS-2) [24]. The second primary outcome measure was the clinician-rated Clinical Global Impression – Improvement scale (CGI) [25].

#### Secondary outcomes

Secondary caregiver-completed outcomes measures included the Repetitive Behavior Scale-Revised (RBS-R) [26], the Aberrant Behavior Checklist – Parent (ABC-P) [27], the Developmental Behavior Checklist – Parent (DBC-P) [28], the Caregiver Strain Questionnaire (CSQ) [29], the PDD Behavior Inventory – Screening Version (PDDBI-SV) [30] and the Short Sensory Profile-2 (SSP-2) [31].

### Statistical analyses

Data were managed using REDCap [32]. Statistical analyses were performed using the Statistical Program for Social Science (SPSS), version 26. A power analysis for a repeated measures design indicated that 87 participants would allow 0.9 power to detect moderate effect sizes at an α-level of 0.05. As used by Sikich et al. (2021) [16], a modified intention-to-treat approach was employed, including all the participants who had undergone randomization, and had both a baseline (Visit 1) and a post-intervention (Visit 3) SRS-2 score. Multiple imputation was used to handle the missing data. The multiple imputations were conducted with the Markov chain Monte Carlo (MCMC) method with 50 iterations using predictive mean matching for missing values. All analyses were two-tailed. Alpha was set at 0.05.

Within the participants who were included in the modified intention-to-treat analysis (*N* = 87), there were 846 missing data points out of 18,531 possible (95.43% completion rate). 50 participants (57.47% of sample) were missing some data. Little’s Missing Completely at Random test indicated randomness in the missing data, χ2(4358) = 332.38, *p* = 1.000.

Analysis of primary and secondary outcomes were conducted on the modified intention-to-treat population and were based on a 2 (Drug; Oxytocin, Placebo) x 4 (Time; baseline wk 0, post-placebo lead in wk 3, post randomized treatment wk 15, follow-up assessment wk 27) x 2 (age group; 3–5 years, 6–12 years) mixed-design ANOVA. Age was entered as a pre-planned factor and our Data Safety Monitoring Board (DSMB) requested that we only recruit for the younger age group following interim analysis. Site was considered as an additional factor but was removed from models due to lack of significance. Greenhouse-Geisser corrections were applied when assumptions of sphericity were violated. For the CGI only, a 2 (Drug; Oxytocin, Placebo) x 3 (Time; post-placebo lead in wk 3, post randomized treatment wk 15, follow-up assessment wk 27) x 2 (age group; 3–5 years, 6–12 years) mixed-design ANCOVA was conducted on improvement scores, with baseline (wk 0) severity scores included as a covariate.

To unpack interaction effects, planned simple contrasts were used, comparing each timepoint to study baseline (Visit 1). For contrasts that reached statistical significance, the sample was first split by age (3–5 years; 6–12 years) and repeated measures ANOVAs were used to compare scores between treatment conditions (oxytocin, placebo). Where appropriate, effect sizes (Cohen’s *d*) are reported, where 0.2 is indicative of a small effect, 0.5 a medium effect and 0.8 a large effect [33].

Sensitivity analyses of the primary outcome included group differences in SRS-2 Total Raw scores (i) separately in the subgroups of participants with a baseline SRS-2 Total Raw score at or above the sample median or with a baseline SRS-2 Total Raw score below the sample median, and (ii) in the per-protocol population (defined as all participants who completed the trial and had SRS-2 data available at each timepoint).

Clinical changes were also evaluated for individual participants using the Reliable Change Index (RCI) [34], which takes into consideration scale reliability of measures used. RCI is equal to an individual’s score before intervention minus their score after an intervention, divided by the standard error of the difference of the measure [34]. If RCI is 1.96 or greater, then the difference is significant. If it is <1.96, RCI is not significant.

Analyses on treatment guess were conducted on participants with available treatment guess data at Visit 3. Chi-square tests were used to compare the proportion of treatment guesses between the oxytocin and placebo conditions. Safety analyses included all participants who were randomized to oxytocin or placebo (*N* = 97). Safety analyses were primarily descriptive; chi-square tests were used to compare the proportion of adverse events potentially related to treatment between the oxytocin and placebo groups.

## Results

### Participants

**Fig. 1:**
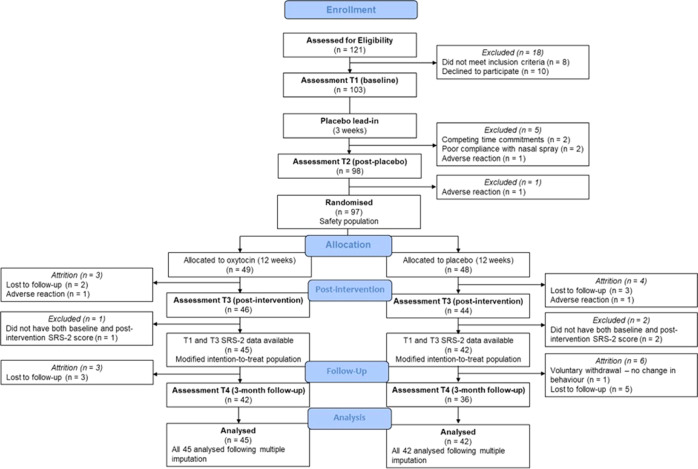
CONSORT diagram of study participants by randomization schedule.

Participants were recruited to each site between April 2017 and February 2020. Caregivers of 331 children expressed interest in this trial. Due to logistical reasons and restraints, the time period for being enrolled in this trial was limited, which impacted on our ability to assess large numbers of participants for eligibility. Further, the broad inclusion criteria meant that there was a high conversion rate from eligibility assessment to study enrollment. In total, 121 children were assessed for eligibility and 103 invited to participate (See Consort Diagram in Fig. 1). A total of 103 children entered the placebo lead in phase and 97 children were then randomized to drug (Oxytocin *N* = 49; Placebo = 48). Eighty-seven participants (Oxytocin = 45; Placebo = 42) had both a baseline and a post-intervention SRS-2 score, with a mean age of 7.27 years (*SD* = 2.69) at baseline. Of these 87 children, 31 were aged 3–5 (Oxytocin *N* = 18; Placebo *N* = 13), while 56 children were aged 6–12 (Oxytocin *N* = 27; Placebo *N* = 29).

### Baseline characteristics

Baseline demographic characteristics and primary outcomes measures of study completers (*N* = 78) were not different from those who did not complete the study (*N* = 9; see Supplementary Table 2). As shown in Table 1, there were no significant differences between the oxytocin and placebo groups on age, gender, full-scale IQ, ADOS classification, psychiatric comorbidities or prescribed medications. Similarly, the oxytocin and placebo groups did not differ on primary or secondary outcome measures at baseline; see Supplementary Table 3).

**Table 1:** Baseline demographic and clinical characteristics across study participants and randomization to drug, *N* = 87.

	Total	Oxytocin	Placebo	Oxytocin-Placebo comparison
	*N* = 87	*N* = 45	*N* = 42	*p*-value
Male, *n* (%)	74 (85.1%)	39 (86.7%)	35 (83.3%)	0.663
Mean age in years (SD, range)	7.27 (2.69, 3.11–12.81)	7.40 (2.95, 3.11–12.81)	7.12 (2.42, 3.20–12.58)	0.628
Mean full-scale IQ (SD, range)^a^	96.75 (15.49, 68–139)	95.47 (16.12, 68–139)	98.00 (14.98. 68–131)	0.502
ADOS classification, *n* (%)^**b**^				
Autism	74 (85.1%)	38 (84.4%)	36 (85.7%)	0.715
Autism spectrum	8 (9.2%)	5 (11.1%)	3 (7.1%)	
Psychiatric comorbidities, *n* (%)				
Any comorbidity	43 (49.4%)	22 (48.9%)	21 (50.0%)	0.918
Sleep disorders	19 (21.8%)	10 (22.2%)	9 (21.4%)	0.929
ADHD	17 (19.5%)	10 (22.2%)	7 (16.7%)	0.514
Anxiety disorders	10 (11.5%)	4 (8.9%)	6 (14.3%)	0.512
Intellectual disability	9 (10.3%)	5 (11.1%)	4 (9.5%)	1.000
Genetic conditions	4 (4.6%)	2 (4.4%)	2 (4.8%)	1.000
Oppositional Disorders	2 (2.3%)	2 (4.4%)	0 (0.0%)	0.495
Depression	2 (2.3%)	0 (0.0%)	2 (4.8%)	0.230
Prescribed medication, *n* (%)				
Any medication	42 (48.3%)	24 (53.3%)	18 (42.9%)	0.328
Melatonin	19 (21.8%)	10 (22.2%)	9 (21.4%)	0.929
Stimulants	10 (11.5%)	5 (11.1%)	5 (11.9%)	1.000
Antipsychotics	10 (11.5%)	5 (11.1%)	5 (11.9%)	1.000
Corticosteroids	8 (9.2%)	6 (13.3%)	2 (4.8%)	0.268
Selective serotonin reuptake inhibitors	8 (9.2%)	3 (6.7%)	5 (11.9%)	0.475
Norepinephrine reuptake inhibitor	1 (1.1%)	1 (2.2%)	0 (0.0%)	1.000
Human growth hormone	1 (1.1%)	1 (2.2%)	0 (0.0%)	1.000

^a^18 participants were unable to complete the Leiter International Performance Scale-Revised due to floor effects. ^b^4 participants did not complete the ADOS-2, but received a DSM-5 diagnosis of autism. 1 participant scored below cut-off on the ADOS-2 but received a DSM-5 diagnosis of autism.

Mid-treatment telephone interviews and post-test assessments indicated participants adhered to the nasal spray administration morning and night 95% of the time on average, and 86% (75/87) of participants reported 90% and above adherence to the routine of delivery.

### Statistical data

Analysis was first conducted to confirm there was no difference across the placebo treatment phases on primary outcome measures (see Supplementary Table 4). This analysis showed no effect of drug condition on placebo response on either measure (*p* > 0.05). Descriptive statistics for primary outcome measures by age group (3–5 years, 6–12 years), along with between- and within-group effect sizes are displayed in Table 2 (see Supplementary Table 5 for descriptive statistics for secondary outcome measures).

**Table 2:** Primary outcomes by treatment condition and age group – whole sample (*N* = 87).

	3–5 years					6–12 years				
Outcome	Oxytocin (*N* = 18)	Placebo (*N* = 13)	Between-group effect size (95% CI)	Within-group effect size^a^ (95% CI)		Oxytocin (*N* = 27)	Placebo (*N* = 29)	Between-group effect size (95% CI)	Within-group effect size^a^ (95% CI)	
	Mean (SD)	Mean (SD)		OXT	PLA	Mean (SD)	Mean (SD)		OXT	PLA
**Primary outcomes**										
**SRS-2**										
Total Raw Score										
Baseline	110.44 (26.64)	92.00 (30.97)	0.65 (−0.10 to 1.36)	–	–	106.11 (25.49)	106.45 (21.13)	−0.01 (−0.54 to 0.51)	–	–
V2	100.11 (20.09)	87.77 (32.14)	0.48 (−0.26 to 1.19)	0.44 (−0.23 to 1.09)	0.13 (−0.64 to 0.90)	97.63 (29.08)	88.87 (25.93)	0.32 (−0.21 to 0.84)	0.31 (−0.23 to 0.84)	0.74 (0.20 to 1.27)
V3	89.89 (20.01)	87.54 (30.31)	0.09 (−0.62 to 0.81)	0.87 (0.17 to 1.53)	0.15 (−0.63 to 0.91)	93.67 (27.82)	84.52 (31.04)	0.31 (−0.22 to 0.83)	0.47 (−0.08 to 1.00)	0.83 (0.28 to 1.35)
V4	96.99 (27.14)	90.70 (24.06)	0.24 (−0.48 to 0.95)	0.50 (−0.17 to 1.15)	0.05 (−0.72 to 0.81)	94.03 (29.25)	86.25 (24.57)	0.29 (−0.24 to 0.81)	0.44 (−0.11 to 0.97)	0.88 (0.33 to 1.41)
CGI^**b**^										
Overall										
Baseline (severity)	4.37 (0.91)	3.89 (0.66)	0.59 (−0.15 to 1.30)	_	_	4.59 (1.08)	4.52 (1.03)	0.07 (−0.46 to 0.59)	–	–
V2 (improvement)	3.33 (0.69)	3.46 (1.27)	−0.13 (−0.84 to 0.58)	_	_	3.81 (0.92)	3.50 (0.69)	0.38 (−0.15 to 0.91)	_	_
V3	3.06 (1.11)	2.85 (1.28)	0.18 (−0.54 to 0.89)	0.29 (−0.37 to 0.94)	0.48 (−0.32 to 1.24)	3.48 (0.70)	3.14 (0.95)	0.41 (−0.13 to 0.93)	0.40 (−0.14 to 0.94)	0.43 (−0.09 to 0.95)
V4	2.89 (1.13)	2.99 (0.94)	−0.09 (−0.81 to 0.62)	0.47 (−0.20 to 1.12)	0.42 (−0.37 to 1.18)	3.75 (0.67)	3.57 (1.01)	0.21 (−0.32 to 0.73)	0.07 (−0.46 to 0.61)	−0.08 (−0.59 to 0.44)

*OXT* oxytocin, *PLA* placebo.

^a^Within-group effect sizes calculated on differences between (i) Baseline and Placebo lead-in; (ii) Baseline and Post-intervention; (iii) Baseline and 3-month follow-up. ^b^Within-group effect sizes for CGI calculated on differences between (i) Placebo lead-in and Post-intervention; (ii) Placebo lead-in and 3-month follow up.

### Primary outcomes

#### Primary outcome 1 – social responsiveness scale (SRS-2) total score

**Fig. 2:**
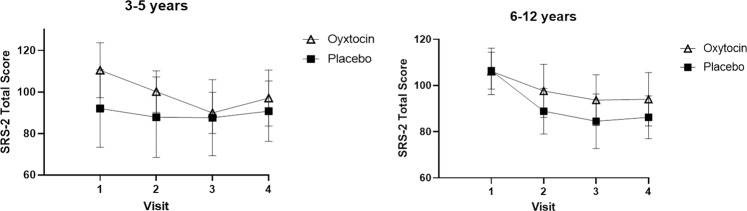
Plotting mean scores on the primary outcome measure (Social Responsiveness Scale-2) separately for younger and older children. Error bars represent 95% confidence intervals.

For SRS-2 Total scores, there was no main effect of treatment condition (*p* = 0.133), and no time by treatment condition interaction, (*p* = 0.686). However, there was a significant 3-way interaction between time, treatment condition and age group, *F*(2.74, 227.39) = 3.15, *p* = 0.028 (see Fig. 2). To break down this interaction, we first used planned contrasts; these contrasts compared total scores in the oxytocin and placebo groups at each timepoint relative to baseline across the 3–5 year and 6–12 year age groups. The contrasts comparing study baseline (V1) to 12-weeks post drug randomization (V3) and study baseline (V1) to 24-weeeks post drug randomization (V4) were statistically significant (*p* = 0.008 and 0.037, respectively).

To further break down this interaction, we used individual repeated measures ANOVAs to look at differences between visits (V1 and V3, V1 and V4) between the oxytocin and placebo conditions separately for each age group. Considering first the 3–5 year age group, there was a significant difference in SRS-2 total scores from V1 to V3; such that participants in the oxytocin condition demonstrated a larger change in scores from baseline to post-treatment (indicating improvement) relative to those in the placebo condition, *F*(1, 29) = 5.25, *p* = 0.029. No such differences were observed in scores from V1 to V4, *F*(1, 29) = 3.33, *p* = 0.068. In the younger group, follow-up exploratory analysis showed that there was greater improvement in the oxytocin condition from V2–V3, in comparison to the placebo condition (t(29) = −1.96, *p* = 0.050; *d* = 0.71). Considering the 6–12 year age group, there was no statistically significant difference in SRS-2 total scores from V1 to V3, *F*(1, 54) = 2.55, *p* = 0.116 or from V1 to V4, *F*(1, 54) = 1.73, *p* = 0.189. Sensitivity analyses showed consistency of these results (See Supplementary Table 6).

#### Primary outcome 2 – clinician global impression–improvement

Considering the second primary outcome, the CGI, there were no significant main effects for time or treatment condition on overall improvement (*p* > 0.365). There was a significant main effect of age group for overall improvement (*p* = 0.004). On average and irrespective of treatment condition or time, younger participants (3–5 years) showed more improvement relative to older participants (6–12 years), as rated by experimenters.

There was no significant three-way interaction between time, treatment condition and age group on the CGI for overall improvement (*p* = 0.809). For details on the proportion of participants who showed improvement on the CGI overall and social communication domains across visits, see Supplementary Tables 7 and 8.

### Secondary outcomes

Overall, there were no significant effects of treatment condition, or interactions between treatment condition, time and age group across the duration of the trial for any total scores of secondary outcomes.

### Clinical data

**Fig. 3:**
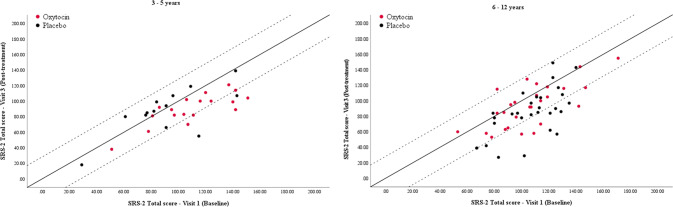
SRS-2 Total scores at Visit 1 (Baseline) and Visit 3 (Post-treatment). The solid diagonal line represents ‘line of no change’ and the dotted lines represent upper and lower Reliable Change Index (RCI) confidence limits. Test-retest reliability = 0.84; s.d. for baseline = 25.47.

Figure 3 shows individual SRS-2 data for participants after oxytocin (red circles) and placebo (black circles). Here, the solid diagonal line represents the ‘line of no change’ between baseline and post-treatment results, whereas the diagonal dotted lines represent the upper and lower RCI confidence limits. Clinically significant improvement was denoted by a RCI > 1.96. Clinically significant deterioration was denoted by a RCI < –1.96.

Inspection of the figure shows that, within the younger age group, 94.4% (17/18) of participants fell at or on the improvement size of the ‘line of no change’ (i.e., RCI = 0) after oxytocin and 38.5% (5/13) of participants fell at or on the improvement size of the ‘line of no change’ (i.e., RCI = 0) after placebo. 27.8% (5/18) participants showed clinically significant improvement after oxytocin, while 15.4% (2/13) showed clinically significant improvement after placebo. No participants showed clinically significant deterioration after oxytocin or placebo.

Within the older age group, 70.4% (19/27) of participants fell at or on the improvement size of the ‘line of no change’ (i.e., RCI = 0) after oxytocin. 82.8% (24/29) of participants fell at or on the improvement size of the ‘line of no change’ (i.e., RCI = 0) after placebo. A total of 18.5% (5/27) participants showed clinically significant improvement after oxytocin, while 31.0% (9/29) showed clinically significant improvement after placebo. 3.7% (1/27) participants showed clinically significant deterioration after oxytocin, while no participants showed clinically significant deterioration after placebo.

### Treatment guess

At Visit 3 (post-treatment) caregivers were asked to guess whether their child had received oxytocin or placebo for the duration of the treatment (see Table 3). A total of 40 (46.5%) guessed their child had received oxytocin, 30 (34.9%) guessed their child had received placebo and 16 (18.6%) reported not knowing which condition their child had been allocated to. There were no significant differences between groups in the correct guess or the percentage that believed they received oxytocin (*p* = 0.173). Treatment guess also did not differ by recruitment site. For treatment guess split by age group (3–5 years, 6–12 years) see Supplementary Tables 9 and 10.

**Table 3:** Treatment Guesses in whole sample (*N* = 86).

	Allocated to oxytocin (*N* = 45)	Allocated to placebo (*N* = 41)
Guessed placebo	17 (37.8%)	13 (31.7%)
Guessed oxytocin	17 (37.8%)	23 (56.1%)
Did not know	11 (24.4%)	5 (12.2%)

### Adverse events

Adverse events are displayed in Table 4. No serious adverse events were considered by the investigators to be related to oxytocin. In the safety population (comprised of the 97 participants randomized to oxytocin or placebo), one participant in the oxytocin group and one participant in the placebo group discontinued the trial regimen due to adverse events. The discontinuation in the oxytocin group was related to increased aggression and the discontinuation in the placebo group was related to a febrile seizure that occurred secondary to infection. For details of specific adverse events in each condition, classified according to terms from the *Medical Dictionary for Regulatory Activities*, version 22.0, see Supplementary Tables 11 and 12. With respect to adverse events that were considered by the investigators to be related to oxytocin or placebo, caregiver reports indicated that children were observed to experience significantly more adverse events when administered placebo as opposed to oxytocin (11/48 vs 2/49), χ^2^(1) = 7.41, *p* = 0.006. The most frequently reported adverse events during the treatment period considered to be related to the nasal spray were increased thirst (5 reports in the placebo condition), nasal discomfort (2 reports in oxytocin condition, 1 report in placebo condition) and rhinorrhoea (3 reports in placebo condition). The most frequently reported adverse events during the placebo lead-in period considered to be related to the nasal spray were epistaxis/nosebleed (5 reports) and increased thirst (4 reports).

**Table 4:** Adverse events (safety population)*.

Adverse event	Oxytocin (*N* = 49)	Placebo (*N* = 48)
Any adverse event—no. of participants (%)	43 (87.8)	41 (85.4)
Maximum intensity of any adverse event in each participant—no. of participants (%)		
Death	0	0
Severe^a^	1 (2.0)	0 (0)
Moderate	5 (10.2)	9 (18.8)
Mild	37 (75.5)	32 (66.7)
Adverse event considered to be related to oxytocin or placebo, according to intensity category – no. of events/total no. (%)		
Severe	0/1 (0)	0 (0)
Moderate^b^	1/6 (16.7)	0/13 (0)
Mild	5/111 (4.5)	26/133 (19.5)
Adverse event leading to withdrawal from trial - no. of participants (%)^c^	1 (2.0)	1 (2.1)
Serious adverse event – no. of participants (%)^d^	1 (2.0)	4 (8.3)

*The safety population included all participants who were randomized to oxytocin or placebo. A list of specific adverse events is provided in Supplementary Tables 11 and 12.

^a^One participant in the oxytocin group experienced a severe allergic reaction to a bee sting.

^b^One participant in the oxytocin group experienced thirst that was rated as moderate severity and this occurred during the placebo lead-in phase.

^c^In the oxytocin group, increased defiant behavior and aggression occurred in one participant. In the placebo group, a febrile seizure due to infection occurred in one participant.

^d^In the oxytocin group, an injured finger occurred in one participant. In the placebo group, two participants lacerated their lips, one had a suicide attempt, and one had an ENT infection and febrile seizure, which were separate and serious adverse events.

## Discussion

The results of this study showed there was no overall benefit of oxytocin treatment for children with autism. Despite this, there was some evidence to suggest that younger children showed greater improvements on social responsiveness at the end of oxytocin treatment, in comparison to placebo. There was no evidence of any benefit from oxytocin treatment to older autistic children, nor was there evidence that observed benefit in the younger age group was maintained following treatment discontinuation. In regards to placebo treatment, this study showed consistent, small to moderate effect sizes of improvement from placebo treatment across measures during the placebo lead-in phase. These effects dissipated during the second phase of placebo treatment (i.e., from Visit 2 to Visit 3 in the placebo group) to support the effectiveness of the placebo washout. In terms of safety, oxytocin nasal spray was well tolerated. There were no serious adverse events linked to oxytocin administration. Interestingly, a greater number of placebo-administered participants reported adverse events in comparison to oxytocin.

While some studies have suggested benefits of oxytocin to younger children [14], rather than older populations [18], this is the first oxytocin study we are aware to demonstrate an age-based interaction effect on outcomes in the trial. In the younger oxytocin condition, young children showed moderate improvement in the placebo lead-in phase and this moderate improvement continued under oxytocin. Whereas, for the placebo group, there was a moderately sized improvement under placebo lead in which did not continue in the second phase of placebo treatment. There has been growing evidence for the potential benefits of social development focused therapies in younger autistic children [8, 35]. Intervention effects may be enhanced at younger ages by causing greater influence on social circuitry [8]. Targeting these circuits for social learning with both therapy and oxytocin may also offer further opportunities to maximize outcomes [8, 36]. Alternatively, shifts on caregiver report measures, like those used in this study, may be easier to observe when focusing on more fundamental skills at younger ages.

Previous studies have also highlighted the potential of placebo-based responses to provide therapeutic outcomes across many clinical conditions [37–39] and also to moderate oxytocin based effects [19]. Meta-analyses in the autism field have further supported the existence of placebo responses of a moderate effect size [40]. Future research is now needed to understand neurobiological, learning and circuitry changes that might be associated with placebo responses in the autism field and methods to potential therapeutically gain from placebo responses. Our study, for example, suggests that short-term placebo effects may provide some benefit in the short-term, but this benefit is unlikely to continue long-term. Studies testing the benefits of short-term placebo treatments seem warranted.

While the safety of oxytocin treatment for children has been debated [41], not only did we find no evidence of deterioration from oxytocin above placebo administration, there were also fewer reported adverse effects in the oxytocin condition. There does, however, remain an urgent need to understand the biological pathways by which oxytocin uptake effects the brain and body. PET imaging studies are required to deliver answers about the bioavailability of oxytocin to the brain and body following intranasal administration. We note that there were many differences between our trial and past trials testing an oxytocin intervention, including trial design, measurement, site, sample size and drug differences. Future clinical trials investigating the efficacy of intranasally administered oxytocin as a treatment needs to standardize procedures for optimal opportunity for delivery, to assess factors that may moderate response to drug, and to collect more objective data to determine response to drug and to change in outcome.

We note limitations of the current study, including a moderate sample size, the inclusion of participants on other psychotropic medications that were stabilized before drug assignment, and reliance on a caregiver and clinician reports as outcome measures. The development of sensitive observational and other markers of change for use in autism clinical trials remains an ongoing priority [42]. We also note the conduct of this study was influenced by both drug supply issues and coronavirus. We had planned to continue recruitment to further consolidate observations in the younger population and to explore markers of response in the younger age group and as recommended by the DSMB. Due to funding limitations, the emergence of coronavirus lockdowns, and the impossibility of obtaining drug supply at the time, we decided study recruitment was no longer feasible, in consultation with the DSMB. The ongoing prioritsiation of sufficient funding for independently led, well- powered, clinical trials is needed in this field where many factors continue to require investigation to determine the therapeutic utility of oxytocin.

In conclusion, the results of this study showed that younger children may benefit from oxytocin nasal spray in comparison to placebo, as indicated by change on caregiver-rated social responsiveness. Oxytocin treatment was found to be well tolerated, with more adverse events reported in the placebo condition. There was evidence of a moderately sized placebo effect across measures in the trial. This study provides one of the first studies in the autism field to demonstrate the benefit of a placebo lead-in phase for clinical trials. There is now a pressing need to conduct larger studies that can definitively test whether younger children benefit from oxytocin, to better disentangle placebo-based effects, and to determine whether oxytocin can be used to enhance social learning circuits. There is also a great need to identify biological pathways underpinning oxytocin uptake, and to identify objective markers of treatment response, in order to better understand how oxytocin may be appropriately used.

## Supplementary information

Consort Checklist

Supplementary Tables
