# Supplementary material for: The effect of oxytocin nasal spray on social interaction in young children with autism: a randomized clinical trial
Source: Mol Psychiatry. 2022 Oct 27;28(2):834–42. doi: 10.1038/s41380-022-01845-8 (PMC9607840; doi:10.1038/s41380-022-01845-8)
Supplement: Supplementary file 2 — Supplementary Tables [file 41380_2022_1845_MOESM2_ESM.docx]

#### Supplementary Table 1. *Schedule of assessments across visits.*

| ***Visit*** | ***0***  ***Diagnostic Assessment*** | ***1***  ***Study Baseline*** | ***2***  ***Post-placebo Lead-in*** | ***3***  ***Post-Intervention*** | ***4***  ***3-month Follow-up*** |
| --- | --- | --- | --- | --- | --- |
| Informed Consent | X |  |  |  |  |
| Exclusion/Inclusion Criteria | X |  |  |  |  |
| Medical History/ Assessment | X |  |  |  |  |
| IQ (Leiter-3) | X |  |  |  |  |
| ASD Diagnosis (ADOS-2) | X |  |  |  |  |
| Rhinometry | X |  |  |  |  |
| Social Interaction/Psychophysiology |  | X | X | X | X |
| Eye-tracking |  | X | X | X | X |
| Caregiver-completed questionnaires |  | X | X | X | X |
| Clinical Global Impression – Improvement Scale |  | X | X | X | X |
| Biomarker collection (blood/saliva) |  | X | X | X | X |
| MRI Scan |  |  | X | X | X |
| IP Dispensing |  | X | X |  |  |
| IP Return |  |  | X | X |  |

#### Supplementary Table 2. *Comparison of baseline characteristics between completers and non-completers*

| **Variable** | **Whole sample** | **Completers** | **Non-completers** | **p-value^*^** |
| --- | --- | --- | --- | --- |
|  | **(n=87)** | **(n=78)** | **(n=9)** |  |
| **Demographics, Mean (SD)** |  |  |  |  |
| Age | 7.27 (2.69) | 7.18 (2.74) | 8.03 (2.26) | .265 |
| Full scale IQ^a^ | 96.75 (15.49) | 96.13 (15.81) | 102.29 (11.79) | .253 |
| ASD severity^b^ | 7.19 (1.51) | 7.16 (1.53) | 7.44 (1.42) | .640 |
| **Concomitant Medications, n (%)** |  |  |  |  |
| None listed | 45 (51.7%) | 40 (51.3%) | 5 (55.6%) | .808 |
| At least one listed | 42 (48.3%) | 38 (48.7%) | 4 (44.4%) |  |
| **Psychiatric Comorbidities, n (%)** |  |  |  |  |
| None listed | 44 (50.6%) | 40 (51.3%) | 4 (44.4%) | .698 |
| At least one listed | 43 (49.4%) | 38 (48.7%) | 5 (55.6%) |  |
| **Gender, n (%)** |  |  |  |  |
| Male | 74 (85.1%) | 66 (84.6%) | 8 (88.9%) | .733 |
| Female | 13 (14.9%) | 12 (15.4%) | 1 (11.1%) |  |
| **SRS-2, Mean (SD)** |  |  |  |  |
| Total – Raw Score | 105.01 (25.47) | 104.63 (25.82) | 108.33 (23.33) | .591 |
| **CGI^c^, Mean (SD)** |  |  |  |  |
| Severity of Illness – Overall | 4.44 (1.00) | 4.42 (0.98) | 4.63 (1.30) | .635 |

*Note:* *Chi-square tests of association conducted for categorical variables; Mann-Whitney U test conducted for continuous variables due to unequal sample sizes. ^a^Full scale IQ scores were not available for 18 participants due to floor effects. ^b^ASD severity scores were not available for 4 participants. ^c^CGI scores were not available for seven participants at baseline.

#### Supplementary Table 3. *Group differences on primary and secondary outcome measures at baseline (Visit 1) – modified intention-to-treat population (N = 87)*

| **Variable** | **Oxytocin**  **(N = 45)** | **Placebo**  **(N = 42)** | **t (*p-*value*)*** | **Effect size**  **(95% CI)** |
| --- | --- | --- | --- | --- |
|  |  |  |  |  |
| **SRS-2** |  |  |  |  |
| Total – Raw Score | 107.84  (25.74) | 101.98  (25.13) | 1.08  (.*286*) | 0.23  (-0.19–0.65) |
| **CGI** |  |  |  |  |
| Severity of Illness – Overall | 4.50  (1.02) | 4.32  (0.97) | 0.81  (.*421*) | 0.18  (-0.24–0.60) |
| **RBS-R** |  |  |  |  |
| Total | 31.71  (16.21) | 29.02  (18.12) | 0.73  (.*466*) | 0.16  (-0.27–0.58) |
| **ABC-P** |  |  |  |  |
| Total | 54.61  (27.24) | 47.88  (26.45) | 1.17  (.*243*) | 0.25  (-0.17–0.67) |
| **DBC-P** |  |  |  |  |
| Total | 54.05  (22.33) | 53.50  (21.59) | 0.12  (.*907*) | 0.03  (-0.40–0.45) |
| **CGS** |  |  |  |  |
| Global Score | 58.11  (17.69) | 59.00  (16.47) | -0.24  (.*809*) | -0.05  (-0.47–0.37) |
| **PDDBI-SV** |  |  |  |  |
| Total Social Deficits – Raw Score | 23.69  (7.51) | 21.88  (8.20) | 1.06  (.*288*) | 0.23  (-0.19–0.65) |
| **SSP-2** |  |  |  |  |
| Sensory Processing Section | 31.30  (9.97) | 34.74  (15.76) | -1.23  (.*220*) | -0.26  (-0.68–0.16) |
| Behavioural Responses Section | 57.99  (16.04) | 57.10  (15.61) | 0.26  (.*794*) | 0.06  (-0.36–0.48) |

*Note:* Data are presented as mean ± SD. Effect sizes reported as Cohen’s *d.*

#### Supplementary Table 4. *Group differences on primary and secondary outcome measures between baseline and post-placebo lead in (change between Visit 1 and Visit 2) – modified intention-to-treat population (N = 87)*

| **Variable** | **Oxytocin**  **(N = 45)** | **Placebo**  **(N = 42)** | **t (*p-*value*)*** | **Cohen’s d** |
| --- | --- | --- | --- | --- |
|  |  |  |  |  |
| **SRS-2** |  |  |  |  |
| Total – Raw Score | -9.22  (17.82) | -13.45  (18.76) | 1.08  (.*282*) | 0.23  (-0.19–0.65) |
| **CGI^a^** |  |  |  |  |
| Global Improvement – Overall | 3.62  (0.86) | 3.49  (0.89) | 0.71  (.*478*) | 0.15  (-0.27–0.57) |
| **RBS-R** |  |  |  |  |
| Total | -3.16  (9.12) | -6.17  (12.18) | 1.31  (*.191*) | 0.28  (-0.14–0.70) |
| **ABC-P** |  |  |  |  |
| Total | -8.02  (19.97) | -11.93  (21.33) | 0.88  (*.378*) | 0.19  (-0.23–0.61) |
| **DBC-P** |  |  |  |  |
| Total | -6.88  (13.42) | -14.89  (18.32) | **2.33**  **(*.020*)** | 0.50  (0.07–0.92) |
| **CGS** |  |  |  |  |
| Global Score | -5.57  (12.75) | -9.27  (15.86) | 1.20  (*.230*) | 0.26  (-0.17–0.68) |
| **PDDBI-SV** |  |  |  |  |
| Total Social Deficits – Raw Score | -1.10  (5.63) | -3.19  (7.20) | 1.46  (.*145*) | 0.32  (-0.10–0.75) |
| **SSP-2** |  |  |  |  |
| Sensory Processing Section | 0.17  (7.78) | -3.67  (10.34) | 1.96  (*.050*) | 0.42  (-0.01–0.84) |
| Behavioural Responses Section | -3.14  (13.47) | -6.58  (13.93) | 1.16  (*.244*) | 0.25  (-0.17–0.67) |

*Note:* Data are presented as mean ± SD. Scores are change scores – calculated as Visit 2 minus Visit 1; negative scores indicate larger change. ^a^CGI Improvement scores are reported as Overall improvement at Visit 2 (post-placebo lead-in) from Visit 1.

#### Supplementary Table 5. *Secondary Outcomes by Treatment Condition and Age Group*

|  | | **3 – 5 years** | | | | | **6 – 12 years** | | | | |
| --- | --- | --- | --- | --- | --- | --- | --- | --- | --- | --- | --- |
| **Outcome** | | **Oxytocin**  **(*N* = 18)** | **Placebo**  **(*N* = 13)** | **Between-Group Effect Size**  **(95% CI)** | **Within-group Effect size^a^**  **(95% CI)** | | **Oxytocin**  **(*N* = 27)** | **Placebo**  **(*N* = 29)** | **Between-Group Effect Size**  **(95% CI)** | **Within-group Effect size^a^**  **(95% CI)** | |
|  | | **Mean**  **(SD)** | **Mean (SD)** |  | **OXT** | **PLA** | **Mean (SD)** | **Mean (SD)** |  | **OXT** | **PLA** |
| ***Secondary Outcomes*** | |  |  |  |  |  |  |  |  |  |  |
| **RBS-R** | |  |  |  |  |  |  |  |  |  |  |
| *Total* | |  |  |  |  |  |  |  |  |  |  |
|  | Baseline | 37.61 (17.51) | 28.62 (18.45) | 0.50  (-0.23–1.21) | – | – | 27.77 (14.28) | 29.21 (18.31) | -0.09  (-0.61–0.44) | – | – |
|  | V2 | 33.89 (15.96) | 23.46 (17.67) | 0.62  (-0.12–1.34) | 0.22  (-0.44–0.87) | 0.29  (-0.50–1.05) | 24.98 (14.68) | 22.57 (16.28) | 0.16  (-0.37–0.68) | 0.19  (-0.34–0.72) | 0.38  (-0.14–0.90) |
|  | V3 | 31.28 (15.77) | 23.08 (16.49) | 0.51  (-0.23–1.22) | 0.38  (-0.29–1.03) | 0.32  (-0.47–1.08) | 27.11 (18.47) | 24.03 (16.66) | 0.18  (-0.35–0.70) | 0.04  (-0.49–0.57) | 0.30  (-0.23–0.81) |
|  | V4 | 33.61 (15.85) | 20.88 (14.59) | 0.83  (0.07–1.55) | 0.24  (-0.42–0.89) | 0.47  (-0.33–1.23) | 22.39 (15.33) | 20.46 (13.88) | 0.13  (-0.39–0.66) | 0.36  (-0.18–0.90) | 0.54  (0.01–1.06) |
| **ABC-P** | |  |  |  |  |  |  |  |  |  |  |
| *Total* | |  |  |  |  |  |  |  |  |  |  |
|  | Baseline | 60.94 (27.94) | 43.77 (21.09) | 0.68  (-0.07–1.39) | – | – | 50.39 (26.45) | 49.72 (28.68) | 0.02  (-0.50–0.55) | – | – |
|  | V2 | 51.17 (26.42) | 40.23 (21.85) | 0.44  (-0.29–1.15) | 0.36  (-0.31–1.01) | 0.16  (-0.61–0.93) | 43.54 (27.35) | 34.03 (21.74) | 0.39  (-0.15–0.91) | 0.25  (-0.28–0.79) | 0.62  (0.08–1.13) |
|  | V3 | 49.39 (25.19) | 37.38 (21.58) | 0.51  (-0.23–1.22) | 0.43  (-0.24–1.09) | 0.30  (-0.48–1.06) | 39.59 (26.78) | 38.38 (27.63) | 0.04  (-0.48–0.57) | 0.41  (-0.14–0.94) | 0.40  (-0.12–0.92) |
|  | V4 | 55.17 (31.40) | 36.22 (17.49) | 0.71  (-0.04–1.43) | 0.19  (-0.46–0.84) | 0.39  (-0.40–1.15) | 42.38 (25.62) | 33.36 (26.66) | 0.34  (-0.19–0.87) | 0.31  (-0.23–0.84) | 0.59  (0.06–1.11) |
| **DBC-P** | |  |  |  |  |  |  |  |  |  |  |
| *Total* | |  |  |  |  |  |  |  |  |  |  |
|  | Baseline | 58.11 (23.49) | 48.31 (22.03) | 0.43  (-0.30–1.14) | – | – | 51.35 (21.54) | 55.83 (21.36) | -0.21  (-0.73–0.32) | – | – |
|  | V2 | 50.63 (16.95) | 43.54 (25.53) | 0.34  (-0.39–1.05) | 0.37  (-0.30–1.02) | 0.20  (-0.58–0.96) | 44.87 (22.23) | 36.40 (19.83) | 0.40  (-0.13–0.93) | 0.30  (-0.25–0.83) | 0.94  (0.39–1.47) |
|  | V3 | 46.56 (18.97) | 39.38 (27.10) | 0.32  (-0.41–1.03) | 0.54  (-0.14–1.19) | 0.36  (-0.42–1.12) | 44.44 (24.23) | 39.21 (22.32) | 0.22  (-0.30–0.75) | 0.30  (-0.24–0.83) | 0.76  (0.22–1.28) |
|  | V4 | 53.22 (21.69) | 32.02 (15.42) | 1.10  (0.31–1.83) | 0.22  (-0.44–0.87) | 0.86  (0.03–1.63) | 42.97 (22.39) | 37.45 (20.14) | 0.26  (-0.27–0.78) | 0.38  (-0.16–0.91) | 0.89  (0.33–1.41) |
| **CGS** | |  |  |  |  |  |  |  |  |  |  |
| *Global Score* | |  |  |  |  |  |  |  |  |  |  |
|  | Baseline | 63.56 (14.07) | 56.23 (18.82) | 0.45  (-0.28–1.16) | – | – | 54.48 (19.13) | 60.24 (15.51) | -0.33  (-0.86–0.20) | – | – |
|  | V2 | 55.17 (16.66) | 50.54 (16.97) | 0.28  (-0.45–0.99) | 0.54  (-0.13–1.20) | 0.32  (-0.47–1.08) | 50.79 (20.93) | 49.36 (16.78) | 0.08  (-0.45–0.60) | 0.18  (-0.35–0.72) | 0.67  (0.14–1.19) |
|  | V3 | 54.33 (15.32) | 53.77 (15.54) | 0.04  (-0.68–0.75) | 0.63  (-0.05–1.28) | 0.14  (-0.63–0.91) | 50.70 (17.21) | 50.76 (17.97) | 0.00  (-0.53–0.52) | 0.21  (-0.33–0.74) | 0.56  (0.03–1.08) |
|  | V4 | 60.50 (19.29) | 53.55 (12.17) | 0.42  (-0.32–1.13) | 0.18  (-0.48–0.83) | 0.17  (-0.61–0.93) | 53.85 (17.61) | 51.46 (16.68) | 0.14  (-0.39–0.66) | 0.03  (-0.50–0.57) | 0.55  (0.01–1.06) |
| **PDDBI-SV** | |  |  |  |  |  |  |  |  |  |  |
| *Total Social Deficits – Raw Score* | |  |  |  |  |  |  |  |  |  |  |
|  | Baseline | 23.75  (7.45) | 22.43 (11.05) | 0.14  (-0.57–0.86) | – | – | 23.65 (7.69) | 21.64 (6.78) | 0.28  (-0.25–0.80) | – | – |
|  | V2 | 24.00  (7.26) | 19.94 (11.00) | 0.45  (-0.28–1.16) | -0.03  (-0.69–0.62) | 0.23  (-0.55–0.99) | 21.65 (7.61) | 18.14 (7.34) | 0.47  (-0.07–0.99) | 0.26  (-0.28–0.79) | 0.50  (-0.03–1.01) |
|  | V3 | 20.56  (6.94) | 21.46 (8.91) | -0.12  (-0.83–0.60) | 0.44  (-0.23–1.09) | 0.10  (-0.68–0.86) | 20.53 (8.89) | 18.02 (9.31) | 0.28  (-0.25–0.80) | 0.38  (-0.17–0.91) | 0.44  (-0.08–0.96) |
|  | V4 | 20.99  (8.52) | 20.90 (7.99) | 0.01  (-0.70–0.72) | 0.34  (-0.32–1.00) | 0.16  (-0.62–0.92) | 21.04 (7.84) | 16.13 (7.44) | 0.64  (0.10–1.17) | 0.34  (-0.21–0.87) | 0.77  (0.23–1.30) |
| **SSP-2** | |  |  |  |  |  |  |  |  |  |  |
| *Sensory Processing Section* | |  |  |  |  |  |  |  |  |  |  |
|  | Baseline | 34.28 (10.96) | 28.31 (10.78) | 0.55  (0.19–1.26) | – | – | 29.31 (8.90) | 37.62 (16.91) | -0.61  (-1.14–-0.06) | – | – |
|  | V2 | 34.67 (10.20) | 28.23 (10.25) | 0.63  (-0.12–1.34) | -0.04  (-0.69–0.62) | 0.01  (-0.76–0.78) | 29.33 (10.36) | 32.34 (10.84) | -0.28  (-0.81–0.25) | 0.00  (-0.54–0.53) | 0.37  (-0.15–0.89) |
|  | V3 | 31.83 (12.88) | 28.38 (10.02) | 0.29  (-0.44–1.00) | 0.20  (-0.45–0.86) | -0.01  (-0.78–0.76) | 31.04 (11.57) | 33.14 (15.01) | -0.16  (-0.68–0.37) | -0.17  (-0.70–0.37) | 0.28  (-0.24–0.79) |
|  | V4 | 35.56 (12.36) | 26.93 (7.61) | 0.81  (0.05–1.53) | -0.11  (-0.76–0.55) | 0.15  (-0.63–0.91) | 28.79 (9.92) | 26.47 (11.07) | 0.22  (-0.31–0.74) | 0.06  (-0.48–0.59) | 0.78  (0.24–1.30) |
| *Behavioural Responses Section* | |  |  |  |  |  |  |  |  |  |  |
|  | Baseline | 63.17 (14.44) | 53.46 (15.03) | 0.66  (-0.09–1.38) | – | – | 54.53 (16.38) | 58.73 (15.85) | -0.26  (-0.78–0.27) | – | – |
|  | V2 | 60.17 (13.85) | 49.92 (15.86) | 0.70  (-0.05–1.41) | 0.21  (-0.45–0.86) | 0.23  (-0.55–0.99) | 51.29 (16.82) | 50.79 (15.44) | 0.03  (-0.49–0.55) | 0.20  (-0.34–0.73) | 0.51  (-0.02–1.02) |
|  | V3 | 55.06 (15.49) | 51.38 (16.96) | 0.23  (-0.49–0.94) | 0.54  (-0.14–1.19) | 0.13  (-0.64–0.90) | 49.85 (17.28) | 47.48 (17.62) | 0.14  (-0.39–0.66) | 0.28  (-0.26–0.81) | 0.67  (0.13–1.19) |
|  | V4 | 62.06 (19.11) | 50.65 (13.88) | 0.67  (-0.08–1.38) | 0.07  (-0.59–0.72) | 0.19  (-0.58–0.96) | 51.46 (15.59) | 46.10 (11.85) | 0.39  (-0.15–0.91) | 0.19  (-0.35–0.72) | 0.90  (0.35–1.43) |

*Note.* OXT=oxytocin; PLA=placebo. Effect sizes reported as Cohen’s *d.* ^a^Within-group effect sizes calculated on differences between (i) Baseline and Placebo lead-in; (ii) Baseline and Post-intervention; (iii) Baseline and 3-month follow-up. ^b^Within-group effect sizes for CGI calculated on differences between (i) Placebo lead-in and Post-intervention; (ii) Placebo lead-in and 3-month follow up.

#### Supplementary Table 6. *SRS-2 Total Raw Scores –Sensitivity Analyses*

| **Outcome** | | **Oxytocin Group** | **Placebo Group** | **Between-Group Effect Size**  **(95% CI)** | **Within-group Effect size^a^**  **(95% CI)** | |
| --- | --- | --- | --- | --- | --- | --- |
|  | | **Mean (SD)** | **Mean (SD)** |  | **OXT** | **PLA** |
|  | |  |  |  |  |  |
| ***Primary Outcome – SRS 2 Total Raw Score*** | | **N = 45** | **N = 42** |  |  |  |
| Baseline | | 107.84  (25.74) | 101.98  (25.13) | 0.23  (-0.19–0.65) | – | – |
| V2 | | 98.62  (25.64) | 88.53  (27.60) | 0.38  (-0.05–0.80) | 0.36  (-0.06–0.77) | 0.51  (0.07–0.94) |
| V3 | | 92.16  (24.81) | 85.45  (30.47) | 0.24  (-0.18–0.66) | 0.62  (0.19–1.04) | 0.59  (0.15–1.02) |
| V4 | | 95.21  (28.15) | 87.62  (24.21) | 0.29  (-0.14–0.71) | 0.47  (0.05–0.88) | 0.58  (0.14–1.01) |
| ***Sensitivity analyses*** | |  |  |  |  |  |
| (i) SRS-2 Total Raw score according to baseline score subgroup | |  |  |  |  |  |
|  | SRS-2 Total Raw score below sample median (< 107) | **N = 22** | **N = 21** |  |  |  |
|  | Baseline | 87.50  (14.92) | 81.43  (16.78) | 0.38  (-0.23–0.98) | – | – |
|  | V2 | 83.36  (24.23) | 70.43  (19.53) | 0.59  (-0.03–1.19) | 0.21  (-0.39–0.79) | 0.60  (-0.03–1.21) |
|  | V3 | 80.00  (21.57) | 70.57  (27.19) | 0.39  (-0.23–0.98) | 0.40  (-0.20–0.99) | 0.48  (-0.14–1.08) |
|  | V4 | 79.81  (25.28) | 78.19  (16.49) | 0.08  (-0.52–0.67) | 0.37  (-0.23–0.96) | 0.19  (-0.42–0.80) |
|  |  |  |  |  |  |  |
|  | SRS-2 Total Raw score above sample median (≥ 107) | **N = 23** | **N = 21** |  |  |  |
|  | Baseline | 127.30  (17.40) | 122.52  (11.25) | 0.32  (-0.28–0.91) | – | – |
|  | V2 | 113.22  (17.28) | 106.63  (22.19) | 0.33  (-0.27–0.92) | 0.81  (0.20–1.40) | 0.90  (0.25–1.52) |
|  | V3 | 103.78  (22.32) | 100.33  (26.45) | 0.14  (-0.45–0.73) | 1.18  (0.53–1.78) | 1.09  (0.42–1.72) |
|  | V4 | 109.95  (22.57) | 97.06  (27.25) | 0.52  (-0.09–1.11) | 0.86  (0.24–1.45) | 1.22  (0.54–1.86) |
| (ii) SRS-2 Total Raw score in per-protocol population | | **N = 42** | **N = 36** |  |  |  |
|  | Baseline | 107.61  (26.66) | 101.53  (25.62) | 0.23  (-0.22–0.68) | – | – |
|  | V2 | 97.85  (26.30) | 89.88  (29.14) | 0.29  (-0.16–0.73) | 0.37  (-0.07–0.80) | 0.42  (-0.05–0.89) |
|  | V3 | 91.29  (25.57) | 85.44  (32.00) | 0.20  (-0.24–0.65) | 0.62  (0.18–1.06) | 0.62  (0.14–1.08) |
|  | V4 | 95.22  (29.47) | 87.09  (26.65) | 0.29  (-0.16–0.73) | 0.44  (0.00–0.87) | 0.55  (0.08–1.02) |

*Note.* OXT=oxytocin; PLA=placebo. Effect sizes reported as Cohen’s *d.* ^a^Within-group effect sizes calculated on differences between (i) Baseline and Placebo lead-in; (ii) Baseline and Post-intervention; (iii) Baseline and 3-month follow-up.

#### Supplementary Table 7. *Clinical Global Impression – Overall Severity and Improvement Separated by Treatment Condition – whole sample (N = 87)*

| **Time point** |  | **Oxytocin**  **(N = 45)** | **Placebo**  **(N = 42)** |
| --- | --- | --- | --- |
|  |  | **n (%)** | **n (%)** |
| Visit 1^a^ | 1 – Normal, not at all ill | 0 (0.0%) | 0 (0.0%) |
|  | 2 – Borderline ill | 0 (0.0%) | 0 (0.0%) |
|  | 3 – Mildly ill | 8 (17.8%) | 8 (19.0%) |
|  | 4 – Moderately ill | 11 (24.4%) | 16 (38.1%) |
|  | 5 – Markedly ill | 14 (31.1%) | 9 (21.4%) |
|  | 6 – Severely ill | 8 (17.8%) | 6 (14.3%) |
|  | 7 – Among the most extremely ill patients | 0 (0.0%) | 0 (0.0%) |
|  |  |  |  |
| Visit 2^b^ | 1 – Very much improved | 0 (0.0%) | 0 (0.0%) |
|  | 2 – Much improved | 3 (6.7%) | 3 (7.1%) |
|  | 3 – Minimally improved | 9 (20.0%) | 12 (28.6%) |
|  | 4 – No change | 30 (66.7%) | 22 (52.4%) |
|  | 5 – Minimally worse | 2 (4.4%) | 2 (4.8%) |
|  | 6 – Much worse | 0 (0.0%) | 0 (0.0%) |
|  | 7 – Very much worse | 0 (0.0%) | 0 (0.0%) |
|  |  |  |  |
| Visit 3 | 1 – Very much improved | 1 (2.2%) | 4 (9.5%) |
|  | 2 – Much improved | 9 (20.0%) | 8 (19.0%) |
|  | 3 – Minimally improved | 11 (24.4%) | 14 (33.3%) |
|  | 4 – No change | 23 (51.1%) | 14 (33.3%) |
|  | 5 – Minimally worse | 1 (2.2%) | 2 (4.8%) |
|  | 6 – Much worse | 0 (0.0%) | 0 (0.0%) |
|  | 7 – Very much worse | 0 (0.0%) | 0 (0.0%) |
|  |  |  |  |
| Visit 4^c^ | 1 – Very much improved | 1 (2.2%) | 1 (2.4%) |
|  | 2 – Much improved | 7 (15.6%) | 7 (16.7%) |
|  | 3 – Minimally improved | 13 (28.9%) | 9 (21.4%) |
|  | 4 – No change | 18 (40.0%) | 15 (35.7%) |
|  | 5 – Minimally worse | 2 (4.4%) | 4 (9.5%) |
|  | 6 – Much worse | 1 (2.2%) | 0 (0.0%) |
|  | 7 – Very much worse | 0 (0.0%) | 0 (0.0%) |

^a^CGI overall severity ratings were missing for 7 participants (4 oxytocin, 3 placebo) at Visit 1.

^b^CGI overall improvement ratings reported as “not assessed” for 2 participants (1 oxytocin, 1 placebo) at Visit 2. CGI overall improvement ratings missing for 2 participants (2 placebo) at Visit 2.

^c^CGI overall improvement ratings missing for 9 participants (3 oxytocin, 6 placebo) at Visit 4.

#### Supplementary Table 8. *Clinical Global Impression – Social Communication Severity and Improvement Separated by Treatment Condition – whole sample (N = 87)*

| **Time point** |  | **Oxytocin**  **(N = 45)** | **Placebo**  **(N = 42)** |
| --- | --- | --- | --- |
|  |  | **n (%)** | **n (%)** |
| Visit 1^a^ | 1 – Normal, not at all ill | 0 (0.0%) | 0 (0.0%) |
|  | 2 – Borderline ill | 0 (0.0%) | 1 (2.4%) |
|  | 3 – Mildly ill | 4 (8.9%) | 6 (14.3%) |
|  | 4 – Moderately ill | 13 (28.9%) | 17 (40.5%) |
|  | 5 – Markedly ill | 13 (28.9%) | 9 (21.4%) |
|  | 6 – Severely ill | 11 (24.4%) | 6 (14.3%) |
|  | 7 – Among the most extremely ill patients | 0 (0.0%) | 0 (0.0%) |
|  |  |  |  |
| Visit 2^b^ | 1 – Very much improved | 0 (0.0%) | 0 (0.0%) |
|  | 2 – Much improved | 5 (11.1%) | 8 (19.0%) |
|  | 3 – Minimally improved | 14 (31.1%) | 12 (28.6%) |
|  | 4 – No change | 25 (55.6%) | 20 (47.6%) |
|  | 5 – Minimally worse | 1 (2.2%) | 0 (0.0%) |
|  | 6 – Much worse | 0 (0.0%) | 0 (0.0%) |
|  | 7 – Very much worse | 0 (0.0%) | 0 (0.0%) |
|  |  |  |  |
| Visit 3 | 1 – Very much improved | 1 (2.2%) | 4 (9.5%) |
|  | 2 – Much improved | 10 (22.2%) | 10 (23.8%) |
|  | 3 – Minimally improved | 21 (46.7%) | 19 (45.2%) |
|  | 4 – No change | 12 (26.7%) | 7 (16.7%) |
|  | 5 – Minimally worse | 1 (2.2%) | 2 (4.8%) |
|  | 6 – Much worse | 0 (0.0%) | 0 (0.0%) |
|  | 7 – Very much worse | 0 (0.0%) | 0 (0.0%) |
|  |  |  |  |
| Visit 4^c^ | 1 – Very much improved | 1 (2.2%) | 0 (0.0%) |
|  | 2 – Much improved | 9 (20.0%) | 10 (23.8%) |
|  | 3 – Minimally improved | 14 (31.1%) | 14 (33.3%) |
|  | 4 – No change | 16 (35.6%) | 11 (26.2%) |
|  | 5 – Minimally worse | 2 (4.4%) | 1 (2.4%) |
|  | 6 – Much worse | 0 (0.0%) | 0 (0.0%) |
|  | 7 – Very much worse | 0 (0.0%) | 0 (0.0%) |

^a^CGI social communication severity ratings were missing for seven participants (4 oxytocin, 3 placebo) at Visit 1.

^b^ CGI social communication improvement ratings missing for 2 participants (2 placebo) at Visit 2.

^c^CGI social communication improvement ratings missing for 9 participants (3 oxytocin, 6 placebo) at Visit 4.

#### Supplementary Table 9. *Treatment Guesses in 3–5 year age group (N = 30)*

|  | Allocated to Oxytocin  (N=18) | Allocated to Placebo  (N=12) |
| --- | --- | --- |
| Guessed Placebo | 6 (33.3%) | 3 (25.0%) |
| Guessed Oxytocin | 10 (55.6%) | 9 (75.0%) |
| Did not know | 2 (11.1%) | 0 (0.0%) |

#### Supplementary Table 10. *Treatment Guesses in 6–12 year age group (N = 56)*

|  | Allocated to Oxytocin  (N=27) | Allocated to Placebo  (N=29) |
| --- | --- | --- |
| Guessed Placebo | 11 (40.7%) | 10 (34.5%) |
| Guessed Oxytocin | 7 (25.9%) | 14 (48.3%) |
| Did not know | 9 (33.3%) | 5 (17.2%) |

The difference in treatment guess conditions was not statistically significant in the 3–5 year age group, χ^2^(2) = 1.93, *p* = .381, or in the 6–12 year age group, χ^2^(2) = 3.46, *p* = .178.

#### Supplementary Table 11. *Incidence of Adverse Events in the Safety Population Classified by MedDRA Term (Placebo lead-in period)*

| **MedDRA Terms** | **Oxytocin (N = 49)** | | | | | **Placebo (N = 48)** | | | | | |
| --- | --- | --- | --- | --- | --- | --- | --- | --- | --- | --- | --- |
| **Preferred system class**  Preferred term | Mild | Mod | Severe | Total AEs | Total Participants (%) | Mild | Mod | Severe | Total AEs | Total Participants (%) |  |
| **Psychiatric** | **12** | **0** | **0** | **12** | **12 (24.49)** | **10** | **0** | **0** | **10** | **9 (18.75)** |  |
| Abnormal sensory level | 2 | 0 | 0 | 2 | 2 (4.08) | 0 | 0 | 0 | 0 | 0 |  |
| Aggression | 2 | 0 | 0 | 2 | 2 (4.08) | 4 | 0 | 0 | 4 | 3 (6.25) |  |
| Agitation | 1 | 0 | 0 | 1 | 1 (2.04) | 0 | 0 | 0 | 0 | 0 |  |
| Anxiety | 0 | 0 | 0 | 0 | 0 | 1 | 0 | 0 | 1 | 1 (2.08) |  |
| Defiant behaviour | 1 | 0 | 0 | 1 | 1 (2.04) | 0 | 0 | 0 | 0 | 0 |  |
| Distractibility | 0 | 0 | 0 | 0 | 0 | 0 | 0 | 0 | 0 | 0 |  |
| Negativism | 0 | 0 | 0 | 0 | 0 | 1 | 0 | 0 | 1 | 1 (2.08) |  |
| Hyperactivity | 3 (1-PR) | 0 | 0 | 3 | 3 (6.12) | 0 | 0 | 0 | 0 | 0 |  |
| Mood swings | 3 | 0 | 0 | 3 | 3 (6.12) | 4 | 0 | 0 | 4 | 4 (8.33) |  |
| **Neurological** | **3** | **0** | **0** | **3** | **3 (6.12)** | **6** | **0** | **0** | **6** | **5 (10.42)** |  |
| Headache | 0 | 0 | 0 | 0 | 0 | 3 | 0 | 0 | 3 | 3 (6.25) |  |
| Insomnia | 1 | 0 | 0 | 1 | 1 (2.04) | 3 (1-PR) | 0 | 0 | 3 | 2 (4.17) |  |
| Terminal insomnia | 1 | 0 | 0 | 1 | 1 (2.04) | 0 | 0 | 0 | 0 | 0 |  |
| Stereotypy | 1 | 0 | 0 | 1 | 1 (2.04) | 0 | 0 | 0 | 0 | 0 |  |
| **General** | **0** | **0** | **0** | **0** | **0** | **2** | **0** | **0** | **2** | **2 (4.17)** |  |
| Energy Increased | 0 | 0 | 0 | 0 | 0 | 1 | 0 | 0 | 1 | 1 (2.08) |  |
| Pyrexia | 0 | 0 | 0 | 0 | 0 | 1 | 0 | 0 | 1 | 1 (2.08) |  |
| **Immune System** | **1** | **0** | **0** | **1** | **1 (2.04)** | **0** | **0** | **0** | **0** | **0** |  |
| Seasonal allergy | 1 | 0 | 0 | 1 | 1 (2.04) | 0 | 0 | 0 | 0 | 0 |  |
| **Renal & Urinary** | **1** | **0** | **0** | **1** | **1 (2.04)** | **4** | **0** | **0** | **4** | **4 (8.33)** |  |
| Frequent urination | 1 | 0 | 0 | 1 | 1 (2.04) | 2 (1-PR) | 0 | 0 | 2 | 2 (4.17) |  |
| Incontinence | 0 | 0 | 0 | 0 | 0 | 2 (1-PR) | 0 | 0 | 2 | 2 (4.17) |  |
| **Injuries & Surgery** | **1** | **0** | **0** | **1** | **1 (2.04)** | **1** | **1** | **0** | **2** | **2 (4.17)** |  |
| Injury – accidental | 1 | 0 | 0 | 1 | 1 (2.04) | 0 | 0 | 0 | 0 | 0 |  |
| Circumcision surgery | 0 | 0 | 0 | 0 | 0 | 0 | 1 | 0 | 1 | 1 (2.08) |  |
| Infection – post surgery | 0 | 0 | 0 | 0 | 0 | 1 | 0 | 0 | 1 | 1 (2.08) |  |
| **Gastrointestinal** | **6** | **1** | **0** | **7** | **7 (14.29)** | **5** | **0** | **0** | **5** | **5 (10.42)** |  |
| Abdominal pain | 0 | 0 | 0 | 0 | 0 | 1 | 0 | 0 | 1 | 1 (2.08) |  |
| Constipation | 2 | 0 | 0 | 2 | 2 (4.08) | 0 | 0 | 0 | 0 | 0 |  |
| Diarrhoea | 2 | 0 | 0 | 2 | 2 (4.08) | 0 | 0 | 0 | 0 | 0 |  |
| Gastroenteritis | 0 | 1 | 0 | 1 | 1 (2.04) | 0 | 0 | 0 | 0 | 0 |  |
| Vomiting | 2 | 0 | 0 | 2 | 2 (4.08) | 2 | 0 | 0 | 2 | 2 (4.17) |  |
| Salivary hypersecretion | 0 | 0 | 0 | 0 | 0 | 1 | 0 | 0 | 1 | 1 (2.08) |  |
| Influenza | 0 | 0 | 0 | 0 | 0 | 1 | 0 | 0 | 1 | 1 (2.08) |  |
| **Metabolic & Nutritional** | **2** | **1** | **0** | **3** | **3 (6.12)** | **3** | **0** | **0** | **3** | **3 (6.25)** |  |
| Decreased appetite | 1 | 0 | 0 | 1 | 1 (2.04) | 0 | 0 | 0 | 0 | 0 |  |
| Thirst | 1 | 1 (1-PR) | 0 | 2 | 2 (4.08) | 3 (3-PR) | 0 | 0 | 3 | 3 (6.25) |  |
| **Skin & Subcutaneous Tissue** | **5** | **0** | **0** | **5** | **5 (10.20)** | **0** | **0** | **0** | **0** | **0** |  |
| Arthropod bite | 1 | 0 | 0 | 1 | 1 (2.04) | 0 | 0 | 0 | 0 | 0 |  |
| Rash | 4 | 0 | 0 | 4 | 4 (8.16) | 0 | 0 | 0 | 0 | 0 |  |
| **Respiratory & Thoracic** | **13** | **0** | **0** | **13** | **13 (26.53)** | **14** | **0** | **0** | **14** | **10 (20.83)** |  |
| Nasal discomfort | 5 (2-PR) | 0 | 0 | 5 | 5 (10.20) | 2 (1-PR) | 0 | 0 | 2 | 2 (4.17) |  |
| Nasopharyngitis | 3 | 0 | 0 | 3 | 3 (6.12) | 1 | 0 | 0 | 1 | 1 (2.08) |  |
| Epistaxis | 1 | 0 | 0 | 1 | 1 (2.04) | 8 (5-PR) | 0 | 0 | 8 | 5 (10.42) |  |
| Rhinorrhoea | 3 | 0 | 0 | 3 | 3 (6.12) | 1 | 0 | 0 | 1 | 1 (2.08) |  |
| Sneezing | 0 | 0 | 0 | 0 | 0 | 2 | 0 | 0 | 2 | 1 (2.08) |  |
| Hand-foot-and-mouth disease | 1 | 0 | 0 | 1 | 1 (2.04) | 0 | 0 | 0 | 0 | 0 |  |
| **Ear & Labyrinth** | **1** | **0** | **0** | **1** | **1 (2.04)** | **0** | **1** | **0** | **1** | **1 (2.08)** |  |
| Earache | 1 | 0 | 0 | 1 | 1 (2.04) | 0 | 0 | 0 | 0 | 0 |  |
| Ear infection | 0 | 0 | 0 | 0 | 0 | 0 | 1 | 0 | 1 | 1 (2.08) |  |
| **Eye** | **1** | **0** | **0** | **1** | **1 (2.04)** | **1** | **0** | **0** | **1** | **1 (2.08)** |  |
| Eye pain | 0 | 0 | 0 | 0 | 0 | 1 | 0 | 0 | 1 | 1 (2.08) |  |
| Lacrimation increased | 1 | 0 | 0 | 1 | 1 (2.04) | 0 | 0 | 0 | 0 | 0 |  |
| **Musculoskeletal** | **1** | **0** | **0** | **1** | **1 (2.04)** | **0** | **0** | **0** | **0** | **0** |  |
| Pain in extremity | 1 | 0 | 0 | 1 | 1 (2.04) | 0 | 0 | 0 | 0 | 0 |  |

*Note.* All AEs included in total AEs. The sum may be greater than the number of participants as some participants had the same AE multiple times during the trial. Participants with the same preferred term and same severity were only counted once. (PR = possibly related)

#### Supplementary Table 12. *Incidence of Adverse Events in the Safety Population Classified by MedDRA Term (Treatment Period)*

| **MedDRA Terms** | **Oxytocin (N = 49)** | | | | | **Placebo (N = 48)** | | | | | |
| --- | --- | --- | --- | --- | --- | --- | --- | --- | --- | --- | --- |
| **Preferred system class**  Preferred term | Mild | Mod | Severe | Total AEs | Total Participants (%) | Mild | Mod | Severe | Total AEs | Total Participants (%) |  |
| **Psychiatric** | **10** | **2** | **0** | **12** | **11 (22.45)** | **16** | **1** | **0** | **17** | **17 (35.42)** |  |
| Abnormal sensory level | 1 | 0 | 0 | 1 | 1 (2.04) | 1 | 0 | 0 | 1 | 1 (2.08) |  |
| Aggression | 1 | 1 | 0 | 2 | 2 (4.08) | 3 (1-PR) | 0 | 0 | 3 | 3 (6.25) |  |
| Anxiety | 1 | 0 | 0 | 1 | 1 (2.04) | 0 | 0 | 0 | 0 | 0 |  |
| Defiant behaviour | 1 | 0 | 0 | 1 | 1 (2.04) | 1 | 0 | 0 | 1 | 1 (2.08) |  |
| Distractibility | 0 | 1 | 0 | 1 | 1 (2.04) | 0 | 0 | 0 | 0 | 0 |  |
| Negativism | 1 | 0 | 0 | 1 | 1 (2.04) | 1 | 0 | 0 | 1 | 1 (2.08) |  |
| Hyperactivity | 0 | 0 | 0 | 0 | 0 | 2 (2-PR) | 0 | 0 | 2 | 2 (4.17) |  |
| Mood swings | 5 | 0 | 0 | 5 | 4 (8.16) | 8 | 0 | 0 | 8 | 8 (16.67) |  |
| Suicide attempt | 0 | 0 | 0 | 0 | 0 | 0 | 1 | 0 | 1 | 1 (2.08) |  |
| **Neurological** | **6** | **1** | **0** | **7** | **6 (12.24)** | **5** | **2** | **0** | **7** | **6 (12.5)** |  |
| Headache | 3 | 0 | 0 | 3 | 2 (4.08) | 1 | 0 | 0 | 1 | 1 (2.08) |  |
| Insomnia | 2 | 0 | 0 | 2 | 2 (4.08) | 2 (1-PR) | 0 | 0 | 2 | 2 (4.17) |  |
| Restlessness | 0 | 1 | 0 | 1 | 1 (2.04) | 0 | 0 | 0 | 0 | 0 |  |
| Stereotypy | 1 | 0 | 0 | 1 | 1 (2.04) | 2 | 0 | 0 | 2 | 2 (4.17) |  |
| Febrile convulsion | 0 | 0 | 0 | 0 | 0 | 0 | 2 | 0 | 2 | 1 (2.08) |  |
| **General** | **0** | **0** | **0** | **0** | **0** | **4** | **0** | **0** | **4** | **4 (8.33)** |  |
| Fatigue | 0 | 0 | 0 | 0 | 0 | 1 | 0 | 0 | 1 | 1 (2.08) |  |
| Pyrexia | 0 | 0 | 0 | 0 | 0 | 3 | 0 | 0 | 3 | 3 (6.25) |  |
| **Immune System** | **0** | **0** | **1** | **1** | **1 (2.04)** | **0** | **0** | **0** | **0** | **0** |  |
| Allergy to arthropod sting | 0 | 0 | 1 | 1 | 1 (2.04) | 0 | 0 | 0 | 0 | 0 |  |
| **Renal & Urinary** | **0** | **0** | **0** | **0** | **0** | **3** | **0** | **0** | **3** | **3 (6.25)** |  |
| Incontinence | 0 | 0 | 0 | 0 | 0 | 3 | 0 | 0 | 3 | 3 (6.25) |  |
| **Injuries & Surgery** | **3** | **0** | **0** | **3** | **3 (6.12)** | **4** | **0** | **0** | **4** | **4 (8.33)** |  |
| Injury – accidental | 0 | 0 | 0 | 0 | 0 | 3 | 0 | 0 | 3 | 3 (6.25) |  |
| Fracture | 2 | 0 | 0 | 2 | 2 (4.08) | 0 | 0 | 0 | 0 | 0 |  |
| Intentional self-injury | 0 | 0 | 0 | 0 | 0 | 1 | 0 | 0 | 1 | 1 (2.08) |  |
| Ear operation | 1 | 0 | 0 | 1 | 1 (2.04) | 0 | 0 | 0 | 0 | 0 |  |
| **Gastrointestinal** | **19** | **0** | **0** | **19** | **16 (32.65)** | **15** | **3** | **0** | **18** | **17 (35.42)** |  |
| Abdominal pain | 0 | 0 | 0 | 0 | 0 | 1 | 0 | 0 | 1 | 1 (2.08) |  |
| Constipation | 6 | 0 | 0 | 6 | 3 (6.12) | 1 | 1 | 0 | 2 | 2 (4.17) |  |
| Diarrhoea | 2 | 0 | 0 | 2 | 2 (4.08) | 5 | 1 | 0 | 6 | 5 (10.42) |  |
| Gastroenteritis | 2 | 0 | 0 | 2 | 2 (4.08) | 1 | 1 | 0 | 2 | 2 (4.17) |  |
| Nausea | 0 | 0 | 0 | 0 | 0 | 1 | 0 | 0 | 1 | 1 (2.08) |  |
| Vomiting | 7 | 0 | 0 | 7 | 7 (14.29) | 3 | 0 | 0 | 3 | 3 (6.25) |  |
| Oropharyngeal pain | 2 | 0 | 0 | 2 | 2 (4.08) | 0 | 0 | 0 | 0 | 0 |  |
| Influenza | 0 | 0 | 0 | 0 | 0 | 3 | 0 | 0 | 3 | 3 (6.25) |  |
| **Metabolic & Nutritional** | **1** | **0** | **0** | **1** | **1 (2.04)** | **5** | **0** | **0** | **5** | **4 (8.33)** |  |
| Thirst | 1 | 0 | 0 | 1 | 1 (2.04) | 5 (5-PR) | 0 | 0 | 5 | 4 (8.33) |  |
| **Skin & Subcutaneous Tissue** | **1** | **0** | **0** | **1** | **1 (2.04)** | **2** | **0** | **0** | **2** | **2 (4.17)** |  |
| Eczema | 0 | 0 | 0 | 0 | 0 | 1 | 0 | 0 | 1 | 1 (2.08) |  |
| Miliaria | 0 | 0 | 0 | 0 | 0 | 1 | 0 | 0 | 1 | 1 (2.08) |  |
| Rash | 1 | 0 | 0 | 1 | 1 (2.04) | 0 | 0 | 0 | 0 | 0 |  |
| **Respiratory & Thoracic** | **22** | **1** | **0** | **23** | **20 (40.82)** | **32** | **4** | **0** | **36** | **30 (62.5)** |  |
| Cough | 0 | 0 | 0 | 0 | 0 | 2 | 0 | 0 | 2 | 2 (4.17) |  |
| Bronchitis | 0 | 0 | 0 | 0 | 0 | 0 | 1 | 0 | 1 | 1 (2.08) |  |
| Nasal congestion | 2 | 0 | 0 | 2 | 2 (4.08) | 0 | 0 | 0 | 0 | 0 |  |
| Nasal discomfort | 2 (2-PR) | 0 | 0 | 2 | 2 (4.08) | 1 (1-PR) | 0 | 0 | 1 | 1 (2.08) |  |
| Nasopharyngitis | 11 | 1 | 0 | 12 | 9 (18.37) | 9 | 1 | 0 | 10 | 7 (14.58) |  |
| Epistaxis | 0 | 0 | 0 | 0 | 0 | 4 (1-PR) | 0 | 0 | 4 | 3 (6.25) |  |
| Pneumonia | 0 | 0 | 0 | 0 | 0 | 1 | 0 | 0 | 1 | 1 (2.08) |  |
| Rhinorrhoea | 6 | 0 | 0 | 6 | 6 (12.24) | 11 (3-PR) | 0 | 0 | 11 | 10 (20.83) |  |
| Sneezing | 0 | 0 | 0 | 0 | 0 | 1 | 0 | 0 | 1 | 1 (2.08) |  |
| Viral infection | 1 | 0 | 0 | 1 | 1 (2.04) | 1 | 0 | 0 | 1 | 1 (2.08) |  |
| Upper respiratory tract infection | 0 | 0 | 0 | 0 | 0 | 2 | 1 | 0 | 3 | 2 (4.17) |  |
| Hand-foot-and-mouth disease | 0 | 0 | 0 | 0 | 0 | 0 | 1 | 0 | 1 | 1 (2.08) |  |
| **Ear & Labyrinth** | **2** | **0** | **0** | **2** | **2 (4.08)** | **1** | **1** | **0** | **2** | **2 (4.17)** |  |
| Ear infection | 2 | 0 | 0 | 2 | 2 (4.08) | 1 | 1 | 0 | 2 | 2 (4.17) |  |

*Note.* All AEs included in total AEs. The sum may be greater than the number of participants as some participants had the same AE multiple times during the trial. Participants with the same preferred term and same severity were only counted once. (PR = possibly related)
